# Immune Control of AIDS Progression by an Adenovirus‐Based Therapeutic Vaccination in Acute Simian Immunodeficiency Virus‐Infected Macaques

**DOI:** 10.1002/mco2.70309

**Published:** 2025-08-01

**Authors:** Yizi He, Chunxiu Wu, Fengling Feng, Zijian Liu, Xugang Zhang, Qing Yang, Zhe Chen, Minjuan Shi, Ziyu Wen, Yichu Liu, Fengyu Hu, Linghua Li, Caijun Sun, Ling Chen, Pingchao Li

**Affiliations:** ^1^ Guangzhou Medical Research Institute of Infectious Diseases Infectious Disease Center Guangzhou Eighth People's Hospital Guangzhou Medical University Guangzhou China; ^2^ State Key Laboratory of Respiratory Disease Institute of Drug Discovery Guangzhou Institutes of Biomedicine and Health Chinese Academy of Sciences Guangzhou China; ^3^ Guangzhou National Laboratory Guangzhou China; ^4^ School of Public Health (Shenzhen) Shenzhen Key Laboratory of Pathogenic Microbiology and Biosafety Shenzhen Campus of Sun Yat‐sen University Shenzhen China; ^5^ Medical College Jinhua Vocational and Technical University Jinhua China; ^6^ University of Chinese Academy of Sciences Beijing China

**Keywords:** adenovirus vector, granulocyte‐macrophage colony‐stimulating factor (GM‐CSF), human immunodeficiency virus (HIV), rhesus macaque, simian immunodeficiency virus (SIV), therapeutic vaccine

## Abstract

Therapeutic vaccinations that enhance human immunodeficiency virus (HIV)‐specific immunity hold promise for reducing reliance on antiretroviral therapy (ART). We previously developed an adenovirus vector‐infected peripheral blood mononuclear cell (AVIP) as a prophylactic strategy that enhanced cellular immunity in macaques and significantly reduced set‐point and peak simian immunodeficiency virus (SIV) loads following SIV challenge. However, its therapeutic efficacy remains to be fully explored. In this study, we improved AVIP by enhancing adenovirus entry into peripheral blood mononuclear cells (PBMCs) through in vitro co‐incubation with granulocyte‐macrophage colony‐stimulating factor (GM‐CSF). We constructed adenoviruses carrying SIV group‐specific antigen (Gag), envelope (Env), and polymerase (Pol) and evaluated the therapeutic potential of autologous AVIP infusion in acute SIV‐infected macaques. Compared with ART alone, AVIP in combination with ART elicited robust cellular immunity against SIV, effectively controlled SIV replication during ART, and delayed viral rebound and acquired immunodeficiency syndrome (AIDS) progression after ART discontinuation. Notably, 80% of macaques in AVIP+ART group maintain plasma virus control for at least 100 days after ART interruption. This sustained viral control is associated with vaccine‐induced Pol‐specific immune responses and reduced CD38 expression on CD8^+^ T cells. These findings support further investigation of AVIP as a therapeutic strategy against acute HIV infection.

## Graphical abstract

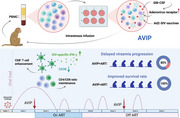

## 1 Introduction

Human immunodeficiency virus (HIV) infection has posed a significant global health challenge ever since acquired immunodeficiency syndrome (AIDS) was first reported in 1981 [1]. As of 2023, approximately 39.9 million people are living with HIV worldwide. While antiretroviral therapy (ART) has proven effective in suppressing viral replication, reducing transmission rates, improving patient quality of life, and reducing AIDS‐related morbidity, it does not eliminate the latent HIV reservoir [2]. Consequently, ART interruption leads to a rapid rebound of plasma HIV load due to the reactivation of persistent latent reservoirs [3]. Furthermore, despite virological suppression achieved through ART, immune recovery is often incomplete [4]. As a result, people infected with HIV must adhere to lifelong ART to prevent disease progression and AIDS. Despite significant advances in ART, there is still no effective prophylactic vaccine against HIV. Therefore, alternative strategies are urgently needed to achieve sustained HIV control without continuous ART or to eradicate viral infection ultimately [5].

Therapeutic vaccination represents a promising approach to address HIV infection, with the potential to achieve prolonged viral suppression or even elimination of viral infection. Unlike prophylactic vaccines, therapeutic vaccines aim to modulate or enhance the host's antiviral immune response in infected individuals, thereby slowing AIDS progression and maintaining undetectable HIV loads without regular ART [6]. While several therapeutic vaccines have been successfully used in cancer treatment [7], the application of therapeutic vaccines against HIV remains under investigation. Although the mechanisms of therapeutic vaccines are still not fully understood, elicited T cell responses are believed to play a crucial role in disease control [7]. Accordingly, the development of therapeutic vaccines against HIV remains a critical priority for controlling HIV infection.

Adenovirus (Ad) vectors are widely used in vaccine development because of their high capacity to deliver foreign genes and elicit strong immunity in mammals [8, 9, 10]. However, the high prevalence of preexisting neutralizing antibodies (NAbs) against common serotypes, such as Ad2 and Ad5, may limit their efficacy. To circumvent preexisting anti‐adenovirus NAbs, various materials have been employed to shield the vector for in vivo delivery [11], and viral vectors have been loaded into target cells via centrifugation [12]. We previously developed a centrifugation‐based immunization strategy termed adenovirus vector‐infected peripheral blood mononuclear cells (AVIPs). This approach involves in vitro infection of autologous peripheral blood mononuclear cells (PBMCs) with Ad‐vectored vaccines, followed by reinfusion into the host, thereby avoiding direct exposure of adenoviral vectors to circulating NAbs. AVIP demonstrated robust immunogenicity and reduced viral set points in simian immunodeficiency virus (SIV)‐challenged rhesus macaques [13]. However, prolonged in vitro culture and excessively high‐intensity centrifugation treatment are not conducive to the state and activity of the cells [14]. Therefore, an alternative strategy was required to optimize our previously reported centrifugation‐based AVIP method.

Granulocyte‐macrophage colony‐stimulating factor (GM‐CSF) plays a critical role in the production, differentiation, and function of granulocytes and macrophages. It is widely used in oncology and immunology, as well as in the clinical treatment of neutropenia, with a good safety record [15, 16]. Previously, we found that co‐incubation with GM‐CSF enhanced Ad vector entry into CD14^+^ cells by upregulating αVβ5 integrin and scavenger receptor A (SR‐A) [17]. These suggest that GM‐CSF may further optimize AVIP efficacy. It is also unknown whether this AVIP strategy could be developed as a therapeutic vaccine. In this study, we optimized the AVIP strategy and evaluated this improved AVIP immunization strategy for its therapeutic potential in acute SIV‐infected rhesus macaques.

## 2 Results

### 2.1 Preincubation With GM‐CSF Enhanced Ad2‐Mediated Gene Expression in PBMCs by Promoting Adenovirus Entry

**FIGURE 1:**
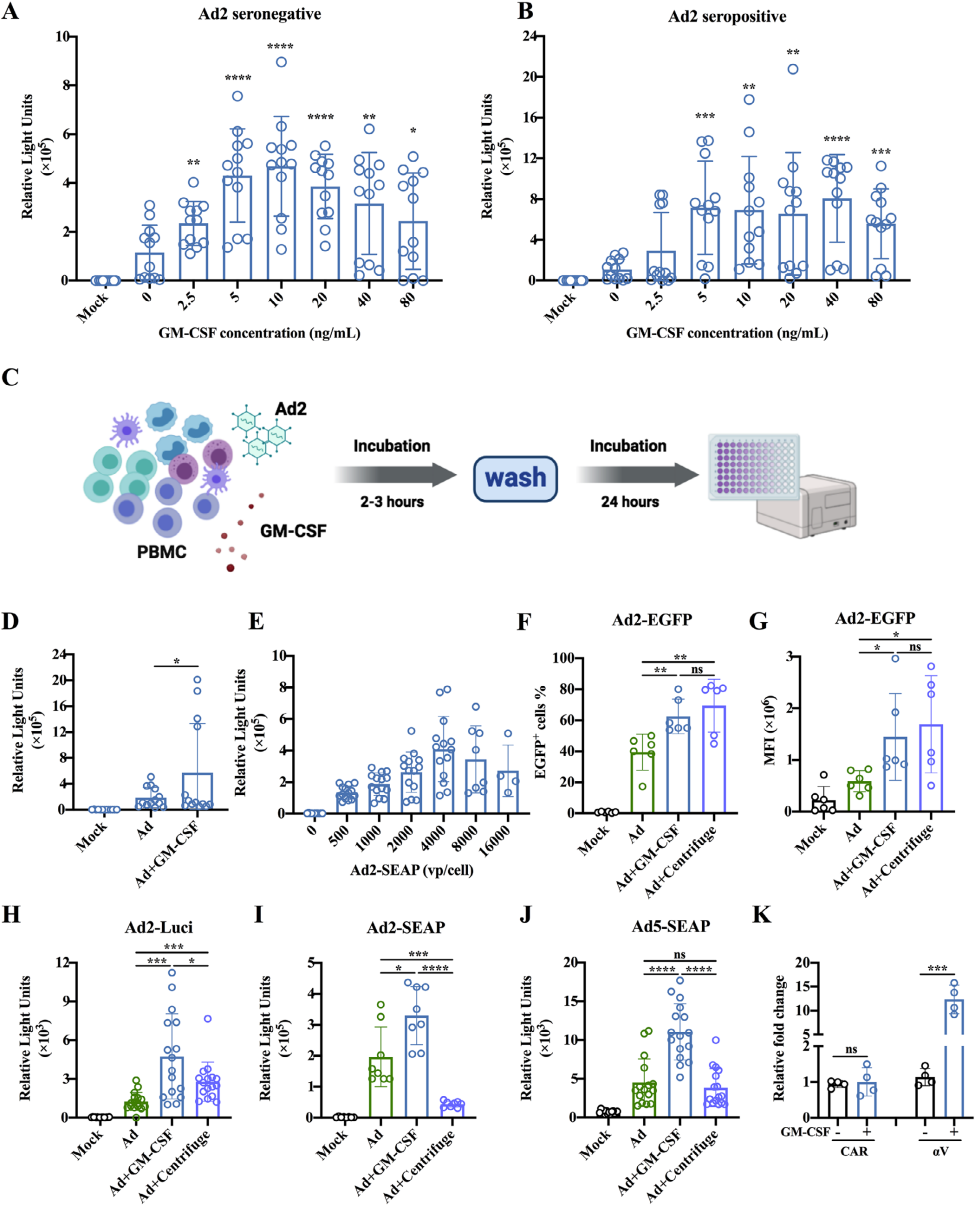
In vitro co‐incubation with GM‐CSF enhanced adenovirus entry into macaque PBMCs. (A,B) Confirmation of the optimal granulocyte‐macrophage colony‐stimulating factor (GM‐CSF) concentration to enhance adenovirus (Ad) entry into macaque peripheral blood mononuclear cells (PBMCs). PBMCs from Ad2‐seronegative (neutralizing antibodies [Nabs] < 18) and Ad2‐seropositive (NAbs > 18) macaques were incubated with 0–80 ng/mL of human GM‐CSF protein and 1250 viral particles (vp)/cell of Ad2 carrying the secreted alkaline phosphatase (SEAP) (Ad2‐SEAP). After 24 h, the relative light unit (RLU) in the cell supernatant was monitored. (C) Schematic representation of PBMCs incubation with GM‐CSF and adenovirus. Macaque PBMCs were incubated with human GM‐CSF (10 ng/mL) and an appropriate dose of reporter adenovirus (Ad2‐EGFP, Ad2‐SEAP, Ad5‐SEAP, and Ad2‐Luci). After 2–3 h of incubation, GM‐CSF and reporter adenovirus were removed by washing, and the expression level of reporter genes was detected after 24 h. Created in BioRender. He, Y. (2025) https://BioRender.com/g34syzi. (D) GM‐CSF enhances the entry of Ad2‐SEAP into macaque PBMCs. Macaque PBMCs were incubated with human GM‐CSF (10 ng/mL) and Ad2‐SEAP for 2–3 h. GM‐CSF and reporter adenovirus were removed by washing, and the RLU in the cell supernatant was monitored after 24 h. (E) Confirmation of the optimal adenovirus dose for infecting macaque PBMCs. Macaque PBMCs were incubated with human GM‐CSF (10 ng/mL) and Ad2‐SEAP (0‐16000 vp/cell) for 2–3 h. GM‐CSF and reporter adenovirus were removed by washing, and the RLU in the cell supernatant was monitored after 24 h. (F,G) Comparison of the efficiency of GM‐CSF co‐incubation with that of centrifugation in promoting the Ad2‐EGFP infection. Macaque PBMCs were incubated with human GM‐CSF (10 ng/mL) and Ad2‐EGFP for 2–3 h. Ad2‐EGFP was added to macaque PBMCs and centrifuged at 1000 g for 1 h. The frequency of EGFP‐positive cells and mean fluorescence intensity (MFI) of EGFP‐positive cells were analyzed after 24 h. (H–J) Comparison of the efficiency of GM‐CSF co‐incubation with that of centrifugation in promoting the adenovirus infection. Macaque PBMCs were incubated with GM‐CSF (10 ng/mL) and Ad2‐Luci, Ad2‐SEAP, and Ad5‐SEAP for 2–3 h, respectively. Ad2‐SEAP, Ad5‐SEAP, and Ad2‐Luci were added to macaque PBMCs and centrifuged at 1000 g for 1 h, respectively. The assay was performed 24 h later. (K) Effect of GM‐CSF on the expression of adenovirus‐associated receptors on macaque PBMCs. Macaque PBMCs were incubated with GM‐CSF (10 ng/mL) and Ad2 for 3 h. The expression of coxsackievirus and adenovirus receptor (CAR) and integrins αV was analyzed using real‐time PCR. Data are represented as the mean ± SD. ns: no significance, *p < 0.05, **p < 0.01, ***p < 0.001, ****p < 0.0001.

We have previously reported that recombinant human GM‐CSF can enhance adenovirus entry into human CD14^+^ cells, particularly those from Ad‐seropositive individuals [17]. To extend these findings to nonhuman primates, we investigated whether GM‐CSF promotes adenovirus entry into PBMCs from Ad2‐seronegative (NAbs < 18) and Ad2‐seropositive (NAbs > 18) macaques. Using Ad2 carrying the secreted alkaline phosphatase (SEAP) gene as a reporter virus, we evaluated adenovirus entry into PBMCs following incubation with varying concentrations of GM‐CSF for 24 h. Our results demonstrated that GM‐CSF facilitated adenovirus entry into PBMCs from both Ad2‐seronegative and Ad2‐seropositive macaques in a dose‐dependent manner (Figure 1A,B). The optimal concentration for promoting adenovirus entry was 10 ng/mL (Figure 1A,B). Interestingly, PBMCs from Ad2‐seropositive macaques exhibited greater susceptibility to Ad2‐SEAP infection than those from Ad2‐seronegative macaques (Figure 1A,B). Furthermore, a brief preincubation period of 2–3 h with GM‐CSF significantly enhanced adenovirus entry into PBMCs (Figure 1C,D).

To optimize the AVIP strategy, we determined that an adenovirus dosage of 4000 viral particles (vp) per cell was optimal for efficient gene transduction in PBMCs (Figure 1E). In addition, we compared the efficiency of GM‐CSF incubation with centrifugation, a previously reported method for enhancing viral entry into cells [18]. We also found that Ad2‐enhanced green fluorescent protein (EGFP) entry into macaque PBMC was significantly promoted by centrifugation, and the optimal centrifugation conditions were 1000 × g for 1 h (Figure S1A–D). Both the frequency of EGFP^+^ cells and the mean fluorescence intensity (MFI) of EGFP^+^ cells were comparable between these two methods (Figure 1F,G). An Ad2 reporter virus carrying a luciferase gene (Ad2‐Luci) showed that incubation of macaque PBMCs with GM‐CSF significantly increased luciferase expression (Figure 1H). Another Ad2 reporter virus carrying SEAP (Ad2‐SEAP) also confirmed that the incubation of macaque PBMCs with GM‐CSF significantly increased SEAP expression in the culture medium (Figure 1I). Surprisingly, centrifugation did not enhance but somewhat attenuated SEAP secretion in PBMCs (Figure 1I). Similar findings were observed with the Ad5‐SEAP reporter virus (Figure 1J), suggesting that centrifugation damages the secretory function of PBMCs.

Mechanistically, GM‐CSF incubation upregulated the expression of integrin αV—an adenovirus receptor—in PBMCs but did not affect coxsackievirus and adenovirus receptor (CAR) (Figure 1K). These findings demonstrate that preincubation of PBMCs with GM‐CSF for 2–3 h is an effective strategy for enhancing adenovirus‐mediated gene transduction in PBMCs.

### 2.2 Improved AVIP Immunization in Combination With ART Effectively Controlled SIV in Acute SIV_mac239_‐Infected Macaques

We have previously demonstrated that the AVIP strategy using Ad5‐vectored vaccines is effective as a prophylactic vaccine because it elicits robust T cell responses and significantly reduces the set point and peak values of SIV loads after SIV challenge [13]. To explore the potential of therapeutic vaccination, we generated three recombinant, replication‐defective type 2 adenoviruses (Ad2‐SIV‐gag, Ad2‐SIV‐env, and Ad2‐SIV‐pol) expressing the SIV_mac239_ group‐specific antigen (Gag), envelope (Env), and polymerase (Pol) proteins, respectively (Figure S2A,B).

We first evaluated the immunogenicity of Ad2‐SIV‐gag, Ad2‐SIV‐env, and Ad2‐SIV‐pol in female C57BL/6 mice, with the Ad2‐empty vector serving as a no‐vaccine control (Figure S3A, Table S1). T cell responses were assessed using interferon (IFN)‐γ enzyme‐linked immunospot (ELISpot) assay and intracellular cytokine staining (ICS). All three SIV vaccines induced significant IFN‐γ‐secreting cells against Gag, Env, and Pol (Figures S3B and S4). ICS revealed increased frequencies of SIV‐specific CD8^+^ and CD4^+^ T cells secreting IFN‐γ, interleukin (IL)‐2, and tumor necrosis factor (TNF)‐α (Figures S3C,D and S5A). In addition, the proliferative capacity of SIV antigen‐stimulated CD8^+^ and CD4^+^ T cells was markedly improved in the vaccinated groups (Figures S3E and S5B). Enzyme‐linked immunosorbent assay (ELISA) revealed that all three Ad2‐SIV vaccines elicited IgG‐binding antibodies against SIV, with significantly higher specific binding antibody titers in the Ad2‐SIV‐env vaccine group compared to the other groups (Figure S3F).

**FIGURE 2:**
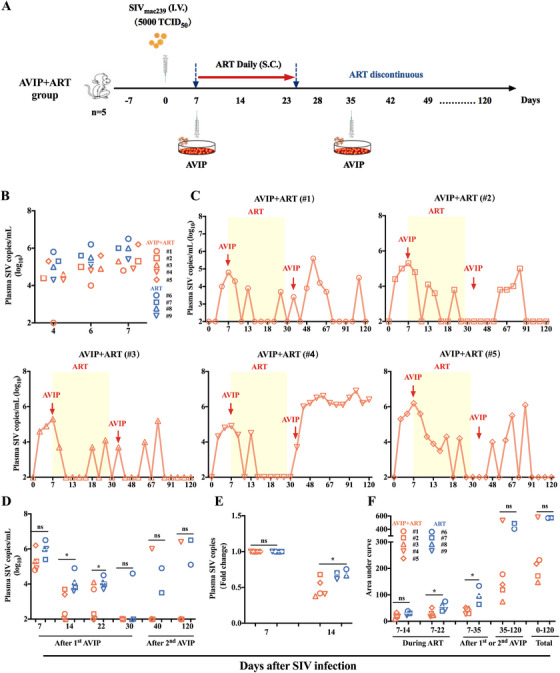
AVIP immunization combined with ART controlled viral load in acute SIV‐infected macaques. (A) Schematic representation of the experimental design of AVIP+ART group. Nine macaques were divided into two groups: five were assigned to AVIP+ART group. All macaques were infected with 5000 tissue culture infectious dose (TCID_50_) simian immunodeficiency virus (SIV) by intravenous injection on Day 0. Antiretroviral therapy (ART) was initiated on Day 0 and lasted 17 days to Day 23. Five macaques in AVIP+ART group were vaccinated with the SIV therapeutic vaccine using this AVIP strategy on Days 7 and 35. (B) Plasma SIV loads of AVIP+ART and ART groups on Days 4, 6, and 7. (C) Plasma SIV viral load of five macaques in AVIP+ART group throughout the study period. (D) Comparison of the plasma SIV load between AVIP+ART and ART groups on Days 7, 14, 22, 30, 40, and 120. (E) Fold change in the plasma SIV load on Day 14 relative to Day 7 in SIV‐infected macaques. (F) Comparison of the area under the curve (AUC) of plasma SIV load in SIV‐infected macaques. Data are represented as the mean ± SD. ns: no significance, *p < 0.05.

**FIGURE 3:**
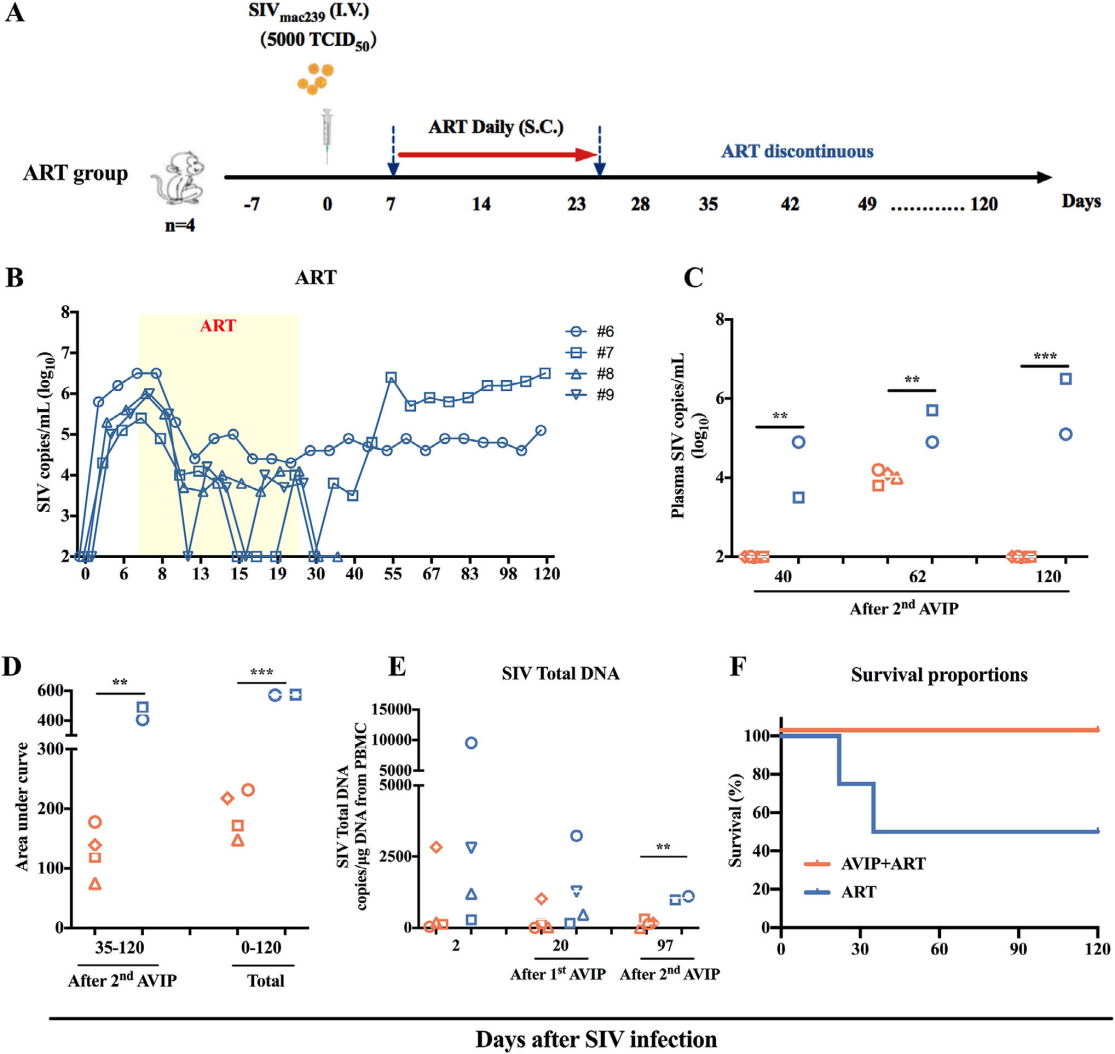
Plasma SIV load and AUC of viral load in ART group macaques. (A) Schematic representation of the experimental design of ART group. Four macaques were assigned to ART group. All macaques were infected with 5000 TCID_50_ SIV by intravenous injection on Day 0. ART was initiated on Day 0 and lasted 17 days to Day 23. (B) Plasma SIV loads of four macaques in ART group throughout the study period. (C) Comparison of plasma SIV loads between AVIP+ART group (except #4) and ART group on Days 40, 62, and 120. (D) Comparison of the AUC of plasma SIV load between AVIP+ART group (except #4) and ART group in SIV‐infected macaques. (E) SIV total DNA in SIV‐infected macaques. Comparison of SIV Total DNA between AVIP+ART group and ART group at Days 2, 20, and 97 (except #4). (F) The survival curves of AVIP+ART and ART groups were analyzed using log‐rank (Mantel‐Cox) tests. Data are represented as the mean ± SD. **p < 0.01, ***p < 0.001, ****p < 0.0001.

To evaluate the therapeutic potential of the improved AVIP immunization strategy, we established an acute SIV_mac239_ infection model in rhesus macaques. Nine macaques were challenged intravenously with 5000 tissue culture infectious dose (TCID_50_) of SIV_mac239_ on Day 0 (Figures 2A and 3A). By Day 4, the plasma viral load was detectable in eight macaques, reaching 4.9 log (4.9 ± 0.6) copies of SIV RNA per milliliter. By Day 6 and Day 7, the plasma viral load in all nine macaques increased to 5.2 log (5.2 ± 0.6) and 5.6 log (5.2 ± 0.6) copies of SIV RNA, respectively (Figure 2B). This means that all nine macaques were successfully infected with SIV. The SIV‐infected macaques were divided into two groups based on sex, age (ranging from 5 to 15 years), body weight (7–17 kg), and the titers of preexisting anti‐adenovirus NAbs (Figure S7A). One group received a combination of AVIP immunization and ART (AVIP+ART group), and the other group received ART alone (ART group) (Table S2).

All macaques received ART (emtricitabine and tenofovir) from Day 7 to 23. In AVIP+ART group, five macaques received AVIP immunization on Days 7 and 35 (Figures 2A and 3A). Plasma SIV loads in the acute SIV_mac239_‐infected macaque model were measured using real‐time quantitative PCR (qPCR) according to methods established in our previous studies [9, 19, 20].

Following ART initiation, plasma viral loads decreased rapidly in all SIV‐infected macaques (Figures 2C and 3B). By Day 11, four out of five macaques (80%) in AVIP+ART group achieved undetectable viral levels (Figure 2C), compared to only one macaque (25%) in ART group (Figure 3B). On Days 14 and 22, plasma viral loads were significantly lower in AVIP+ART group than in ART group (Figure 2D). The fold decrease in viral load from Day 7 to 14 was greater in AVIP+ART group (Figure 2E). In addition, the area under the curve (AUC) for plasma SIV loads during ART (from Day 7 to 22) and after the first AVIP immunization (from Day 7 to 35) was lower in AVIP+ART group (Figure 2F). These results suggest that during ART, AVIP immunization significantly inhibited SIV replication in macaques with acute SIV infection.

After ART discontinuation on Day 23, viral rebound was continuously monitored. AVIP+ART group exhibited a longer interval to viral rebound than ART group. In AVIP+ART group, four of five macaques (except #4) showed viral blips for at least 100 days after ART discontinuation and did not reach a set point at the final time point (Figures 2C and 3B). AVIP+ART group (except #4) maintained significantly lower viral loads on Days 40, 62, and 120 than ART group (Figures 2D and 3C). Meanwhile, the AUC for SIV loads from Day 35 to 120 and from Day 0 to 120 in AVIP+ART group (except #4) was significantly lower than in ART group (Figures 2F and 3D). qPCR analysis of SIV reservoirs revealed reduced total DNA levels in both AVIP+ART and ART groups. At 2.5 months (Day 97 post‐ART interruption), SIV total DNA levels were significantly lower in AVIP+ART group compared to ART group (except #4) (Figure 3E).

Notably, two macaques (#9 and #8) in ART group died on Days 22 and 44 (50% survival rate). In contrast, all macaques in AVIP+ART group survived for at least 120 days (100% survival rate) (Figure 3F). These findings suggest that the improved AVIP combined with ART effectively inhibits viral replication and delays AIDS progression after ART discontinuation in acute SIV‐infected macaques.

### 2.3 Improved AVIP Immunization in Combination With ART Enhanced SIV‐Specific T Cell Immunity in Acute SIV_mac239_‐Infected Macaques

**FIGURE 4:**
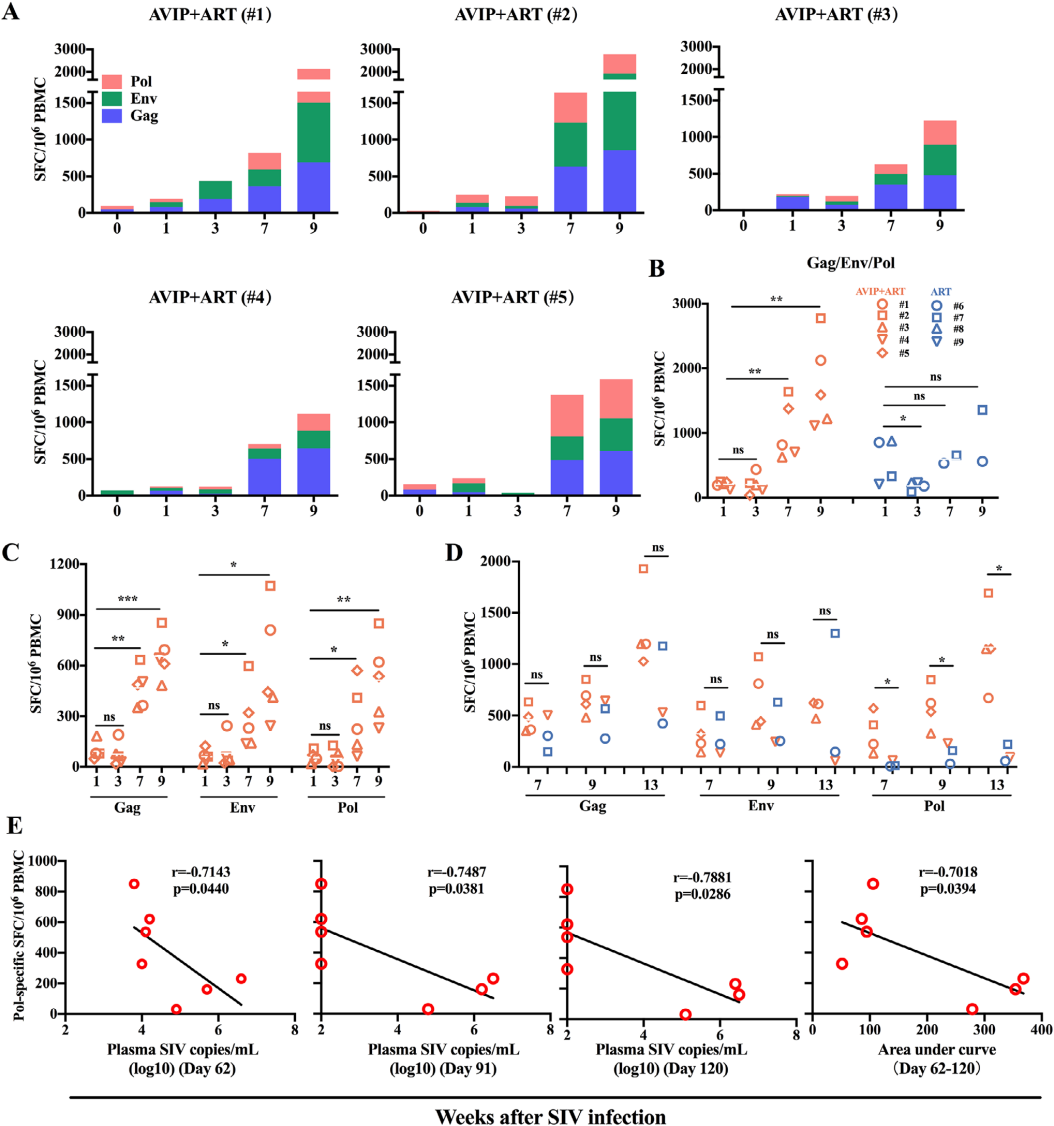
Specific T cell immune responses elicited by AVIP immunization combined with ART in SIV‐infected macaques. (A) Total SIV‐specific spot‐forming cells (SFCs) per million PBMCs at different time points after SIV infection in AVIP+ART group. (B) Comparison of total SIV‐specific SFCs per million PBMCs at different time points in SIV‐infected macaques. (C) Comparison of group‐specific antigen (Gag)‐, envelope (Env)‐, and polymerase (Pol)‐specific SFCs per million PBMCs at different time points in AVIP+ART group. (D) Comparison of Gag‐, Env‐, and Pol‐specific SFCs per million PBMCs between macaques with better control of plasma viral load (#1, #2, #3, #5) and those with poor control of plasma viral load (#4, #6, #7) after ART interruption. (E) Correlations between Pol‐specific SFCs at Week 7 versus plasma SIV load at Days 62, 91, and 120, and versus the AUC of plasma SIV load from days 62 to 120. Data are represented as the mean ± SD. ns: no significance, *p < 0.05, **p < 0.01, ***p < 0.001.

**FIGURE 5:**
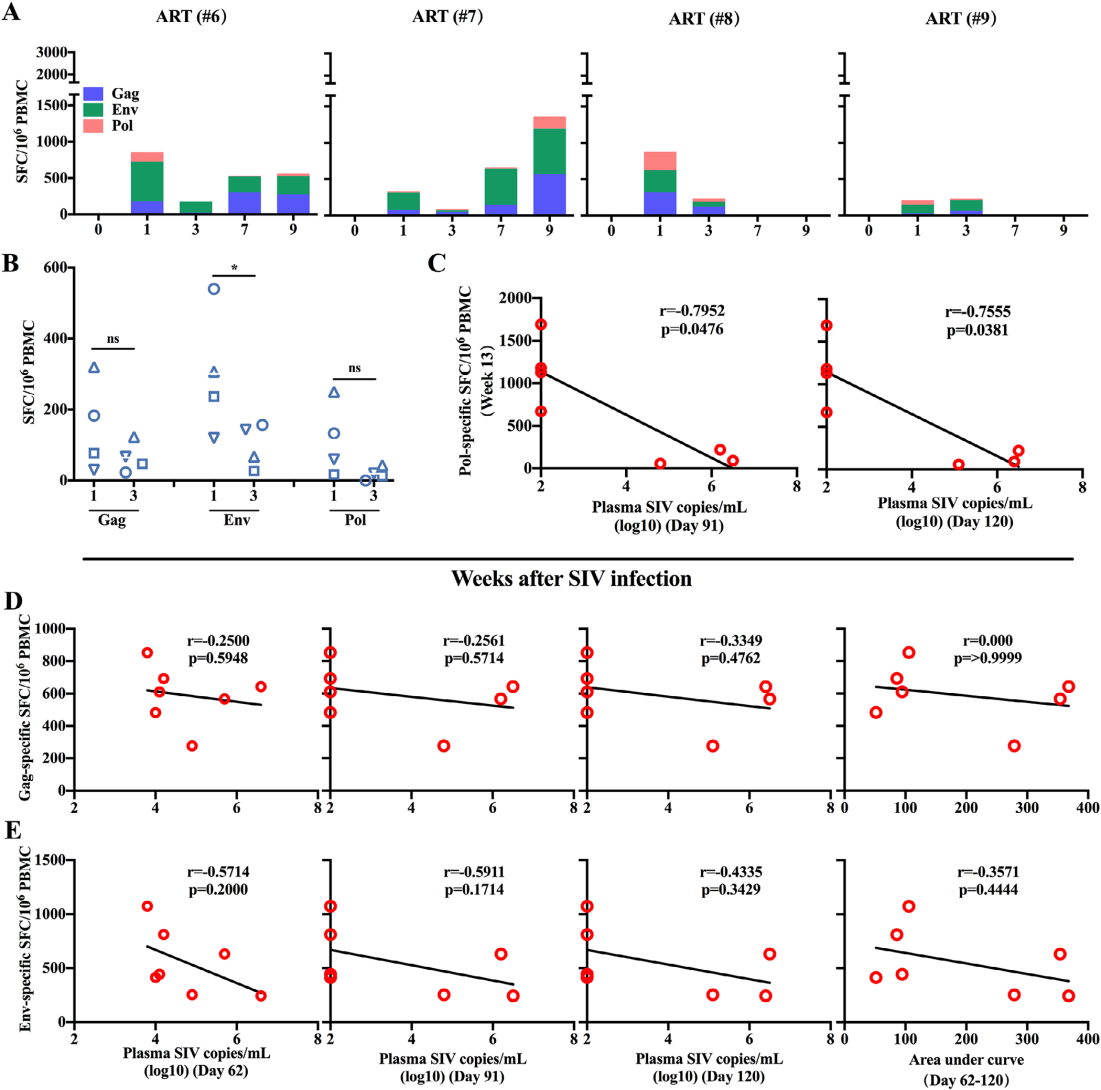
SIV‐specific T cell response and correlation analysis in ART group macaques. (A) Total SIV‐specific SFCs per million PBMCs at different time points after SIV infection in ART group. (B) Comparison of Gag‐, Env‐, and Pol‐specific SFCs per million PBMCs between Week 1 and Week 3 in ART group. (C) Correlation between Pol‐specific SFCs at Week 13 versus plasma SIV load on Days 91 and 120. (D) Correlation between Gag‐specific SFCs at Week 7 versus plasma SIV load on Days 62, 91, and 120, and versus AUC of plasma SIV load from Days 62 to 120. (E) Correlation between Env‐specific SFCs at Week 7 versus plasma SIV load on Days 62, 91, and 120, and versus AUC of plasma SIV load from Days 62 to 120. Data are represented as the mean ± SD. ns: no significance, *p < 0.05.

Specific T cell immunity is crucial for controlling and managing HIV infection [21]. To evaluate the effects of the improved AVIP strategy on SIV‐specific immunity, we conducted longitudinal assessments of IFN‐γ responses using ELISpot assays at multiple time points following AVIP immunization (Figures 4A and 5A). In ART group, the frequency of antigen‐specific spot‐forming cells (SFCs) declined significantly by Week 3 (2 weeks post‐AVIP immunization). In contrast, no such decline was observed in AVIP+ART group (Figures 4A,B and 5A). This suggests that improved AVIP immunization effectively preserves T cell immunity during early HIV/SIV infection. Specifically, Env‐specific but not Gag‐ or Pol‐specific IFN‐γ‐secreting PBMCs were significantly reduced in ART group (Figure 5B), highlighting the protective role of AVIP in maintaining Env‐specific immune responses. Two weeks after the second AVIP immunization (Week 7), all three SIV antigens (Gag, Env, and Pol) elicited robust IFN‐γ‐secreting PBMCs in AVIP+ART group, which were sustained at higher levels than ART group (Figure 4A–C). Preexisting anti‐Ad2 NAbs in SIV‐infected macaques were found not to affect the immune effect of this AVIP immunization (Figure S7B,C).

We further sought to determine whether the T cell responses elicited by AVIP immunization were correlated with better control of SIV infection. Macaques with better plasma viral load control post‐ART discontinuation (#1, #2, #3, and #5) exhibited significantly stronger specific T cell immunity against Pol, but not against Env or Gag, at Weeks 7, 9, and 13 compared to those with poor viral control (#4, #6, and #7) (Figure 4D). Importantly, Pol‐specific T cell responses at Week 7 (i.e., 2 weeks after the second AVIP immunization) were negatively correlated with plasma SIV loads at Days 62, 91, and 120, and with AUC of viral load from Day 62 to 120 (Figure 4E). Similarly, Pol‐specific T cell responses at Week 13 (i.e., 8 weeks after second AVIP immunization) also showed a negative correlation with viral loads at Days 91 and 120 (Figure 5C). In contrast, Gag‐ and Env‐specific T cell responses induced by AVIP immunization did not correlate significantly with plasma SIV loads or the AUC during the same period (Figure 5D,E). These findings demonstrate that an improved AVIP immunization strategy, when combined with ART, enhances SIV‐specific T cell immunity, particularly against the Pol antigen, and correlates with better viral control following ART withdrawal.

### 2.4 Improved AVIP Immunization in Combination With ART Reduced Excessive Activation of CD8^+^ T Cells in Acute SIV‐Infected Macaques

**FIGURE 6:**
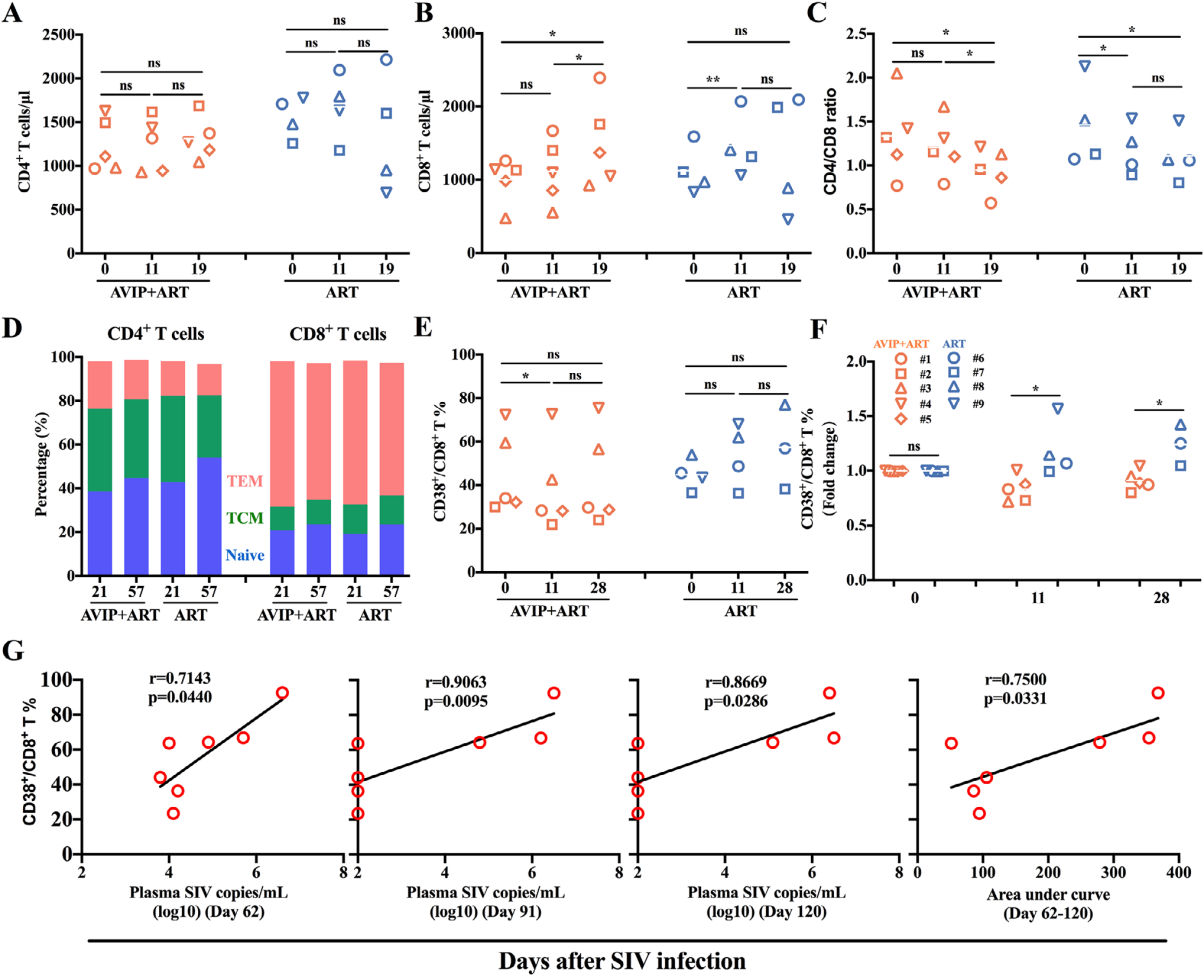
Immune cell activation induced by AVIP immunization combined with ART in SIV‐infected macaques. (A–C) The kinetics of CD4^+^ T cell counts, CD8^+^ T cell counts, and CD4/CD8 ratio in AVIP+ART group and ART group during the study period. (D) The percentage of naïve (CD28^+^CD95^−^), and central memory T cells (TCM) (CD28^+^CD95^+^), and effector memory T cells (TEM) (CD28^−^CD95^+^) on CD4^+^ and CD8^+^ T cells in AVIP+ART group and ART group. (E) The percentage of CD38^+^/CD8^+^ T cells was compared at different time points between AVIP+ART group and ART group. (F) Fold change of CD38^+^/CD8^+^ T cell percentage at Days 11 and 28 relative to Day 0 in SIV‐infected macaques. (G) Correlations between CD38^+^/CD8^+^ T cell percentage at Week 7 versus plasma SIV load at Days 62, 91, and 120, and versus AUC of plasma SIV load from Days 62 to 120. Data are represented as the mean ± SD. ns: no significance, *p < 0.05, **p < 0.01.

We investigated whether improved AVIP immunization affects the quantity and quality of T cells in acute SIV‐infected macaques using flow cytometry (Figure S6A). No significant differences were observed between AVIP+ART and ART groups in the CD4/CD8 ratio, CD8^+^ T cell counts, and CD4^+^ T cell counts over time (Figure 6A–C). During ART, AVIP immunization did not significantly alter CD4^+^ T cell counts but induced a notable increase in CD8^+^ T cells on Day 19 (Figure 6A,B). On Day 11, the CD4/CD8 ratio was significantly lower in ART group compared to the baseline (Day 0). In contrast, this ratio remained stable in AVIP+ART group (Figure 6C). From Day 19 onward, the CD4/CD8 ratio in AVIP+ART group began below baseline levels (Figure 6C). These findings suggest that AVIP immunization combined with ART helps preserve the CD4/CD8 ratio and prevents its rapid decline in acute SIV‐infected macaques.

To further evaluate T cell subset changes, we analyzed naïve T cells, effector memory T cells (TEM), and central memory T cells (TCM) based on CD28 and CD95 expression levels following AVIP immunization (Figure S6B). No significant differences were observed between AVIP+ART and ART groups regarding the percentages of naïve, TEM, and TCM cells within CD4^+^ and CD8^+^ T cell populations (Figure 6D).

Enhanced CD38 expression on CD8^+^ T cells may be associated with increased immune system activation and the progression of HIV infection [22, 23]. Interestingly, AVIP immunization during ART significantly reduced CD38 expression on CD8^+^ T cells, particularly observed on Day 12. This decrease was not observed in ART group (Figure 6E). The fold change in CD38 expression relative to baseline was significantly lower in AVIP+ART group compared to ART group on Days 12 and 28 (Figure 6F).

Further analysis revealed a positive correlation between CD38 expression levels on CD8^+^ T cells at Week 7 and plasma SIV loads at Days 62, 91, and 120 (Figure 6G). In addition, CD38 expression levels at Week 4 were positively associated with the AUC of plasma SIV load from Day 62 to 120 (Figure 6G). Notably, the two macaques in ART group that died from infection exhibited higher CD38 levels on their CD8^+^ T cells before death compared to other infected macaques (Figure 6E). In contrast to CD38, the expression of programmed cell death protein 1 (PD‐1) on CD8^+^ T cells did not differ significantly between AVIP+ART group and ART group. Furthermore, AVIP immunization did not influence PD‐1 expression levels (Figure S8A). The fold change in PD‐1 expression relative to baseline also showed no significant differences between the two groups on Days 12 and 28 (Figure S8B).

Taken together, improved AVIP immunization strategy, when combined with ART, attenuates the HIV/SIV infection‐induced decrease in the CD4/CD8 ratio and reduces excessive activation of CD8^+^ T cells in acute SIV‐infected macaques.

## 3 Discussion

ART has successfully transformed HIV infection from a fatal disease into a manageable chronic condition. However, there is currently no effective vaccine or complete cure for HIV infection. Therefore, alternative strategies are required to achieve viral eradication or sustained ART‐free remission [2]. Therapeutic vaccination represents a promising approach to enhance or restore the host immune response against HIV [6, 7, 24]. In this study, we demonstrated that autologous infusion of AVIP therapeutic vaccine combined with ART elicited robust SIV‐specific T cell immune responses, prevented a rapid decline in the CD4/CD8 ratio, reduced CD8^+^ T cell overactivation, accelerated viral suppression, and delayed post‐ART viral rebound and AIDS progression in acute SIV‐infected macaques.

Our investigation of SEAP secretion revealed that centrifugation diminished supernatant SEAP activity, suggesting that centrifugation forces impair cell secretory function while enhancing adenoviral entry. Similar to our study, previous studies have also reported that centrifugation, especially at higher centrifugal forces or longer holding times, causes severe sublethal damage to cells, including membrane integrity loss and viability reduction [25, 26]. In contrast, GM‐CSF‐mediated adenovirus entry maintains cell morphology and activity by upregulating the expression of adenovirus receptors.

Our improved AVIP approach minimizes NAbs interference and blood component interactions by delivering vaccine antigens directly into CD14^+^ monocytes/macrophages. SIV vaccine antigens can be expressed, processed, and antigenically presented directly in antigen‐presenting cells (APC). Compared to our previous centrifugation‐based method [13], our AVIP approach by incubation with GM‐CSF improved adenovirus entry without compromising APC secretory function, potentially enhancing vaccine‐induced immune responses. While the precise mechanism of GM‐CSF‐mediated integrin upregulation remains unclear, this approach demonstrates superior APC activity over the centrifugation‐based method.

Due to their critical roles in viral replication, we selected the SIV structural proteins Gag, Env, and Pol as vaccine antigens. Gag‐specific responses disrupt viral assembly [27], Env‐targeted immunity blocks viral entry [28], and Pol‐specific T cells inhibit enzymatic function essential for viral replication [29]. Our previous study demonstrated that AVIP delivery of these vaccine antigens induced protective T cell responses in macaques [13]. Based on these findings, we evaluated the therapeutic potential of an improved AVIP strategy in acute SIV‐infected macaques.

Early ART initiation during acute HIV infection minimizes immune damage and reduces viral reservoirs but fails to eliminate infected cells [30]. While ART alone rapidly suppressed SIV replication, it also reduced HIV‐specific T cell responses within 1‐2 weeks [31]. In this study, we also found that Env‐specific T cell immunity in acute SIV‐infected macaques was significantly reduced after 2 weeks of ART initiation compared to pre‐ART. This is likely due to the rapid suppression of viral replication by ART, which reduces antigenic stimulation and leads to a contraction of virus‐specific T cell responses. In contrast, AVIP+ART group maintained stable Env‐specific T cell responses during the same period, correlating with faster early viral control. Similar to our findings, a previous study showed that early Env‐specific CD8^+^ T cell immunity suppressed viral replication in acute chimeric simian/human immunodeficiency virus (SHIV) controller macaques [32].

The robust Gag‐ and Env‐specific T cell responses observed following SIV infection may mask AVIP immunization‐induced responses. In contrast, the Pol antigen is generally less immunogenic during natural infection. Thus, vaccine‐elicited Pol‐specific T cell responses may be more distinguishable and play a more prominent role in viral control [29, 33, 34]. Our study suggests that AVIP immunization elicited strong Pol‐specific T cell responses sustained during ART and contributed to delayed viral rebound after ART interruption. The magnitude of T cell immunity against Pol negatively correlated with viral load control after ART interruption. Previously, our study indicated that mucosal vaccination with a replicating vaccinia virus‐based SIV vaccine, followed by boosting with an adenovirus‐based SIV vaccine, elicited protective immunity correlated with CD8^+^ T cell immunity against Gag and Pol [35]. Consistently, a previous study demonstrated that the antiviral activity of CD8^+^ T cells correlated with the rapid presentation of Pol and Gag epitopes in HLA‐B^*^2705‐positive HIV patients [29]. These findings suggest that the induction of specific T cell immunity, particularly against subdominant antigens such as Pol, may be highly beneficial in designing therapeutic vaccines to eliminate infected cells and inhibit the expansion of latent viral reservoirs.

CD38 is widely distributed and expressed on peripheral blood T, NK, and B cells [23]. The upregulated expression of CD38 on CD8^+^ T cells during HIV infection correlates with immune system activation, HIV disease progression, and poor prognosis [23, 36]. We recognize that the lack of an untreated control group limits our ability to comprehensively analyze the individual contributions of ART and AVIP immunization to changes in CD38^+^CD8^+^ T cell levels. However, previous studies showed that ART significantly reduces CD38 expression on CD8^+^ T cells in HIV‐infected individuals by inhibiting viral replication [23, 37, 38]. In this study, we observed that AVIP+ART group exhibited a more significant reduction in CD38^+^CD8^+^ T cells than ART alone group, suggesting that AVIP immunization may contribute to immune modulation beyond ART. This may be attributed to AVIP immunization enhancing virus‐specific T cell responses, improving viral control, and reducing immune activation. In this study, our results also demonstrated that the expression level of CD38 on CD8^+^ T cells was positively correlated with plasma SIV load. This also suggests that reducing the level of abnormal immune activation of T cells during HIV infection, especially in the early or acute phase of infection, is critical and may directly affect patient prognosis. However, the mechanism by which this AVIP immunization reduces T cell overactivation in acute SIV‐infected macaques needs further investigation.

Notably, macaque #4 in AVIP+ART group exhibited a distinct response, with viral loads rebounding within approximately a week of ART discontinuation and rapidly reaching set point. Throughout the course of AVIP+ART, we observed that the level of SIV‐specific immune response, particularly Pol‐specific immune response, was lowest among the five macaques. Our and others' previous studies showed a significant negative correlation between Pol‐specific immune response and viral control [29, 35]. In addition, we observed that among the five macaques in AVIP+ART group, macaque #4 had the highest level of CD8^+^ cells expressing CD38, a well‐recognized marker of T cell exhaustion, with a significant positive correlation between its expression level and viral load. Accordingly, the failure of virus control in macaque #4 might be caused by impaired immune function of T cells.

The SIV‐infected macaque model remains indispensable for HIV cure research, as humanized mouse models cannot fully simulate human immune responses. Our acute SIV‐infected macaque model simulates key aspects of acute HIV infection and enables evaluation of the antiviral efficacy of AVIP combined with ART. We believe that the therapeutic strategy of AVIP combined with ART in SIV‐infected macaques may be translated to HIV patients.

While our findings demonstrate that AVIP combined with ART strategy delays AIDS progression in acute SIV‐infected macaques, this study has several limitations. First, the small cohort size warrants cautious interpretation. Second, the absence of healthy controls limits comparative analysis. Third, the efficacy of lymphoid tissue penetration and chronic infection remains unaddressed. Future studies should evaluate follicular trafficking of vaccine‐elicited responses in SIV infection models. Future studies should also explore the long‐term durability of immune responses and the mechanistic basis underlying viral control following treatment interruption.

In conclusion, this proof‐of‐concept study confirms that the GM‐CSF‐mediated adenovirus‐based SIV vaccine delivery strategy in combination with ART enhances antiviral immunity during acute infection. This strategy delays viral rebound and disease progression following ART by maintaining specific T cell responses and reducing immune activation. While further validation is needed, these findings validate that AVIP combined with ART is a promising candidate strategy for the functional cure of AIDS.

## 4 Materials and Methods

### 4.1 Recombinant Adenovirus SIV Vaccines

Recombinant adenovirus SIV vaccines carrying antigens Gag, Pol, and Env were constructed as previously described [19, 39]. SIV genes *gag*, *env*, and *pol* were cloned into pGA1 plasmids to generate pGA1‐SIVgag, pGA1‐SIVenv, and pGA1‐SIVpol. These plasmids underwent homologous recombination with the pAd2‐empty vector to produce pAd2‐SIVgag, pAd2‐SIVenv, and pAd2‐SIVpol plasmids. The recombinant adenoviruses were rescued in Trex293 cells, and their expression of target proteins was confirmed via Western blot. Purification was performed using cesium chloride density gradient centrifugation. Virus concentrations were determined using UV spectrophotometry, while viral titers were quantified using the Spearman‐Kärber method and expressed as TCID_50_/mL.

### 4.2 PBMC Isolation and Adenovirus Infection

PBMCs were isolated from fresh macaque blood samples using OptiPrep gradient solution (Axis‐Shield, Norway) according to the manufacturer's instructions.

For in vitro infection, PBMCs were treated with human GM‐CSF (R&D Systems, USA) at concentrations ranging from 2.5 to 80 ng/mL and exposed to reporter adenoviruses (Ad2‐EGFP, Ad2‐SEAP, Ad5‐SEAP, or Ad2‐Luci). After a 2–3 h incubation, cells were washed to remove unbound GM‐CSF and adenovirus. Reporter protein expression was assessed 24 h later.

For centrifugal infection, PBMCs were treated with the appropriate dose of reporter adenovirus by centrifugation at 1000 g for 1 h at room temperature. Infected cells were cultured in a complete RPMI 1640 medium for 24 h.

### 4.3 Establishment of Acute SIV_mac239_‐Infected Macaque Model

Macaques (*n* = 9) were intravenously infected with SIV_mac239_ at a dose of 5000 TCID_50,_ as previously reported [20]. Blood samples were collected at various time points post‐infection to monitor plasma SIV load, and two independent detections of viral RNA confirmed successful infection. Infected macaques received subcutaneous injection of ART with emtricitabine (20 mg/kg/day) and tenofovir (30 mg/kg/day).

### 4.4 Animal Vaccination

Female C57BL/6 mice (6 weeks) were used and received intramuscular vaccinations with SIV vaccines (Ad2‐SIV‐gag, Ad2‐SIV‐env, and Ad2‐SIV‐pol) at a dose of 1 × 10^10^ vp per animal. Two weeks later, serum and spleen samples were collected for immunological analysis.

Nine acute SIV_mac239_‐infected macaques were divided into AVIP+ART group (*n* = 5) and ART group (*n* = 4). In AVIP+ART group, autologous PBMCs were treated in vitro with GM‐CSF and 10¹¹ vp of adenovirus SIV vaccines (Ad2‐SIVgag, Ad2‐SIVenv, and Ad2‐SIVpol) for 2–3 h. On Days 7 and 35 post‐infection, macaques in AVIP+ART group were vaccinated intramuscularly using the AVIP strategy. Blood samples and PBMCs were collected at multiple time points to evaluate immunological and virological responses.

### 4.5 SIV Peptide Pools

SIV_mac239_ peptide pools were obtained from the National Institutes of Health (NIH) HIV Reagent Program. These peptides consist of 15 amino acids and 11 overlapping residues, covering the total sequence of SIV_mac239_ Gag, Env, and Pol proteins. Peptide pools were dissolved in dimethyl sulfoxide (DMSO, Sigma‐Aldrich, USA) as stock solutions at a final concentration of 0.4 mg/mL.

### 4.6 ELISpot

IFN‐γ ELISpot was processed as previously reported [40, 41]. ELISpot plates (Merck Millipore, Germany) were coated overnight with anti‐monkey IFN‐γ antibody (U‐CyTech, Netherlands) at 4°C. Plates were washed and blocked with R10 medium (RPMI 1640 supplemented with 10% FBS, 1% penicillin‐streptomycin, 0.05 mM 2‐mercaptoethanol, 1 mM sodium pyruvate, 2 mM L‐glutamine, and 10 mM HEPES) for 2 h at 37°C. PBMCs were seeded at a density of 200,000 cells per well. Diluted DMSO, SIV peptides, and positive stimulus concanavalin A (ConA) were added. The plate was incubated at 37°C, 5% CO_2_, for 24 h. The monkey IFN‐γ ELISpot detection antibody (U‐CyTech) was added. The streptomycin‐conjugated alkaline phosphatase (BD Pharmingen, USA) was added and incubated at 37°C for 2 h. 5‐bromo‐4‐chloro‐3‐indolyl phosphate/nitro blue tetrazolium (BCIP/NBT) substrate solution (Pierce, USA) was added for 10 min. Spots were counted using an ELISpot reader (Bioreader 4000, Germany). Data are reported as SFCs per million PBMCs.

### 4.7 ELISA

SIV‐specific IgG antibodies in serum were detected via ELISA. SIV lysates were coated onto 96‐well plates at 1 µg/mL overnight at 4°C. Plates were blocked with phosphate‐buffered saline with Tween 20 (PBST) containing 5% skimmed milk for 2 h at room temperature. Serially diluted mouse sera were added and incubated for 2 h. Goat anti‐mouse secondary antibody was then added, followed by 3,3′,5,5′‐tetramethylbenzidine (TMB) substrate (Thermo Fisher, USA). After a 5–10 min incubation at 37°C in the dark, the reaction was stopped, and optical density (OD) was measured at 450 nm.

### 4.8 SEAP‐Based Ad2‐Neutralizing Antibody Assay

NAbs against Ad2 were measured as previously reported [42]. In brief, 293 cells were cultured in 96‐well plates for 24 h. A serial dilution of macaque sera was incubated with Ad2‐SEAP at 37°C at 4 × 10^6^ vp per well for 1 h. Next, the mixtures were added to 293 cells and incubated at 37°C for 24 h. Cell‐free culture supernatants were collected to detect SEAP activity by a Phospha‐Light System (Thermo Fisher). The relative light unit (RLU) was monitored using a luminometer (MLX Microtiter, USA). The NAb titers against Ad2 were calculated using a dilution of 50% inhibited RLU values.

### 4.9 ICS

ICS was performed as previously reported [19]. Briefly, cells were adjusted to a concentration of 2 × 10^6^ per well and added into 96‐well plates. Cells were incubated with SIV peptide pools, DMSO, and positive control phorbol 12‐myristate 13‐acetate (PMA, 40 ng/mL) and Ionomycin (Ion, 1 µg/mL) for 2 h. Brefeldin A (BD Biosciences, USA) was added, and cells were cultured for 12–16 h. Surface antibodies were applied (Table S3), then fixation and permeabilization using the Cytofix/Cytoperm kit (BD Biosciences). Cells were stained with anti‐IFN‐γ, IL‐2, and TNF‐α antibodies. Cell samples were analyzed by an LSR Fortessa flow cytometer (BD Biosciences). The flow cytometry data were analyzed by FlowJo software (Tree Star, USA).

### 4.10 Flow Cytometry Analysis

For the detection of naïve T cells, TEM, and TCM, PBMCs were incubated with surface antibodies targeting CD3, CD4, CD8, CD95, and CD28 for 30 min (Table S3). The percentages of naïve (CD28^+^CD95^−^), TCM (CD28^+^CD95^+^), and TEM (CD28^−^CD95^+^) on CD4^+^ and CD8^+^ T cells in AVIP+ART and ART groups were detected using an LSR Fortessa flow cytometer (BD Biosciences) and analyzed with FlowJo software (Tree Star).

### 4.11 T Cell Proliferation

PBMCs were resuspended in RPMI 1640 medium and labeled with carboxyfluorescein diacetate succinimidyl ester (CFSE) (1 µM) (Molecular Probes, USA). Cells were incubated with SIV peptide pools for 5 days. Surface antibodies against CD3, CD4, and CD8 were added before analysis on an LSR Fortessa flow cytometer (BD Biosciences). Data were analyzed using FlowJo software (Tree Star) (Table S3).

### 4.12 Plasma SIV RNA Quantification

Plasma SIV RNA was quantified as previously reported [19, 20, 41]. The QIAamp Viral RNA Mini kit (Qiagen, Germany) was used to extract RNA according to the manufacturer's protocol and quantified using the QuantiTect SYBR Green RT‐PCR Kit (Qiagen). SIV RNA quantities were determined using a standard curve from SIV gag RNA standards. All samples were run and analyzed using a CFX96 Real‐Time PCR system (Bio‐Rad, USA).

### 4.13 T Cell Count Assays

Blood samples were collected according to standard protocols [20]. For T cell counts, T cells in the blood were quantified using BD Trucount Absolute Count Tubes (BD Biosciences). Antibodies against CD45‐PE, CD3‐APC, CD4‐FITC, and CD8‐PercP (all from BD Biosciences) were used to identify different T cell populations. Cells were lysed with FACS Lysing solution (BD Biosciences), and samples were analyzed using a BD Accuri C6 Plus flow cytometer (BD Biosciences). Data were processed using FlowJo software (Tree Star).

### 4.14 Statistical Analysis

GraphPad Prism 8 software (GraphPad Software, USA) was used for statistical analysis. Unpaired students’ *t‐*tests were used to compare AVIP+ART and ART groups. Paired students’ *t‐*tests were used to compare different time points within AVIP+ART and ART groups. A significant difference was set at *p* < 0.05.

## Author Contributions

P.L., L.C., and C.S. supervised and designed the study. Y.H., C.W., and F.F. performed original draft preparation, literature collection, and rewriting. F.F., Y.H., P.L., C.W., Z.L., X.Z., Q.Y., Z.C., M.S., Z.W., and Y.L. performed experiments. Y.H. and F.F. analyzed data. L.C., C.S., P.L., L.L., and F.H. contributed in reagents and materials. All authors have read and agreed to publish this manuscript.

## Ethics Statement

Mice (C57BL/6) were housed at the experimental animal center of Guangzhou Institutes of Biomedicine and Health (GIBH), Chinese Academy of Sciences. Chinese rhesus macaques (*Macaca mulatta*) were housed at Guangdong Landau Biotechnology Co. Ltd. All experimental protocols were approved by the Institutional Animal Care and Use Committee (IACUC) of GIBH (No. 2019088). All procedures were carried out under the supervision of trained professional staff.

## Conflicts of Interest

The authors declare no conflicts of interest.

## Supporting information



Supporting Information
Figure S1. Effect of different centrifugation conditions on adenovirus entry into macaque PBMCs.
Figure S2. Construction of Ad2‐SIV‐gag, Ad2‐SIV‐env, and Ad2‐SIV‐pol vaccines.
Figure S3. Evaluation of immunogenicity of adenovirus vectored SIV vaccines in mice.
Figure S4. Representative images of IFN‐γ ELISpot
Figure S5. Representative images of ICS and CFSE
Figure S6. The flow cytometry gating strategy.
Figure S7. Correlations between pre‐existing anti‐Ad2 NAbs versus the SIV‐specific immune responses
Figure S8. The expression of PD‐1 on CD8+ T cells in SIV‐infected macaques.
Table S1. Vaccination in mice
Table S2. Information of rhesus macaques
Table S3. Antibody information for flow cytometry analysis

## Data Availability

Additional datasets generated and analyzed during the current study are available from the corresponding author upon reasonable request.
