# Supplementary material for: Immune Control of AIDS Progression by an Adenovirus‐Based Therapeutic Vaccination in Acute Simian Immunodeficiency Virus‐Infected Macaques
Source: MedComm (2020). 2025 Aug 1;6(8):e70309. doi: 10.1002/mco2.70309 (PMC12314542; doi:10.1002/mco2.70309)
Supplement: Supplementary file 1 — Supporting Information Figure S1. Effect of different centrifugation conditions on adenovirus entry into macaque PBMCs. Figure S2. Construction of Ad2‐SIV‐gag, Ad2‐SIV‐env, and Ad2‐SIV‐pol vaccines. Figure S3. Evaluation of immunogenicity of adenovirus vectored SIV vaccines in mice. Figure S4. Representative images of IFN‐γ ELISpot Figure S5. Representative images of ICS and CFSE Figure S6. The flow cytometry gating strategy. Figure S7. Correlations between pre‐existing anti‐Ad2 NAbs versus the SIV‐specific immune responses Figure S8. The expression of PD‐1 on CD8+ T cells in SIV‐infected macaques. Table S1. Vaccination in mice Table S2. Information of rhesus macaques Table S3. Antibody information for flow cytometry analysis [file MCO2-6-e70309-s001.docx]

**Supplementary Information**

**Title:** **Immune control of AIDS progression by an adenovirus-based therapeutic vaccination in acute simian immunodeficiency virus-infected macaques**

**Author:** Yizi He^1^^,2#^, Chunxiu Wu^4#^, Fengling Feng^3,5#^, Zijian Liu^2,6^, Xugang Zhang^2,6^, Qing Yang^2^, Zhe Chen^2^, Minjuan Shi^3^, Ziyu Wen^3^, Yichu Liu^2^, Fengyu Hu^1^, Linghua Li^1*^, Caijun Sun^3*^, Ling Chen^1,2,4*^, Pingchao Li^2,6*^

**Affiliations:**

^1^Guangzhou Medical Research Institute of Infectious Diseases, Infectious Disease Center, Guangzhou Eighth People’s Hospital, Guangzhou Medical University, Guangzhou, China

^2^State Key Laboratory of Respiratory Disease, Institute of Drug Discovery, Guangzhou Institutes of Biomedicine and Health, Chinese Academy of Sciences, Guangzhou, China.

^3^School of Public Health (Shenzhen), Shenzhen Key Laboratory of Pathogenic Microbiology and Biosafety, Shenzhen Campus of Sun Yat-sen University, Shenzhen, China.

^4^Guangzhou National Laboratory, Guangzhou, China.

^5^Medical College, Jinhua Vocational and Technical University, Jinhua, China.

^6^University of Chinese Academy of Sciences, Beijing, China.

*Corresponding authors: Pingchao Li, li_pingchao@gibh.ac.cn; Ling Chen, [chen_ling@gibh.ac.cn](mailto:chen_ling@gibh.ac.cn); Caijun Sun, [suncaijun@mail.sysu.edu.cn](mailto:suncaijun@mail.sysu.edu.cn); Linghua Li, llheliza@126.com

^#^These authors contributed equally: Yizi He, Chunxiu Wu, Fengling Feng

**
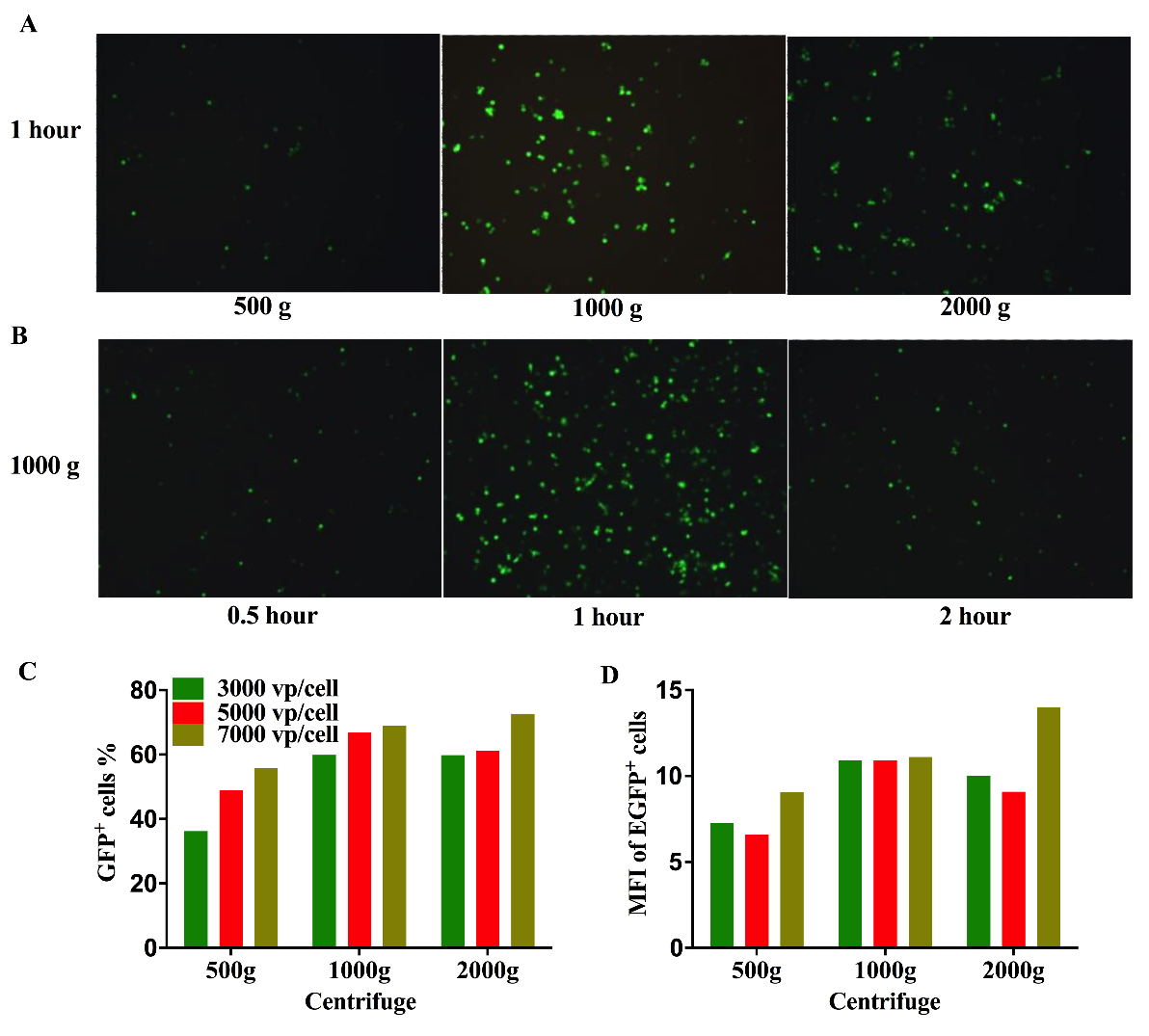
**

**Figure S1. Effect of different centrifugation conditions on adenovirus entry into macaque PBMCs.**

1. Effect of different centrifugation speeds on adenovirus entry into macaque peripheral blood mononuclear cells (PBMCs). Ad2-enhanced green fluorescent protein (EGFP) was added to macaque PBMCs, followed by centrifugation at different centrifugation speeds (500×g, 1000×g, and 2000×g) for 1 hour, and EGFP expression was monitored 24 hours later.
2. Effect of different centrifugation times on adenovirus entry into macaque PBMCs. Ad2-EGFP was added to macaque PBMCs, followed by centrifugation at 1000×g for different centrifugation times (0.5, 1, and 2 hours), and EGFP expression was monitored 24 hours later.

**(C-D)** Ad2-EGFP (3000, 5000, and 7000 viral particles (vp)/cell) was added to macaque PBMCs, followed by centrifugation at 1000×g for 1 hour, respectively. The frequency of EGFP-positive cells and mean fluorescence intensity (MFI) of EGFP-positive cells were analyzed after 24 hours.

**
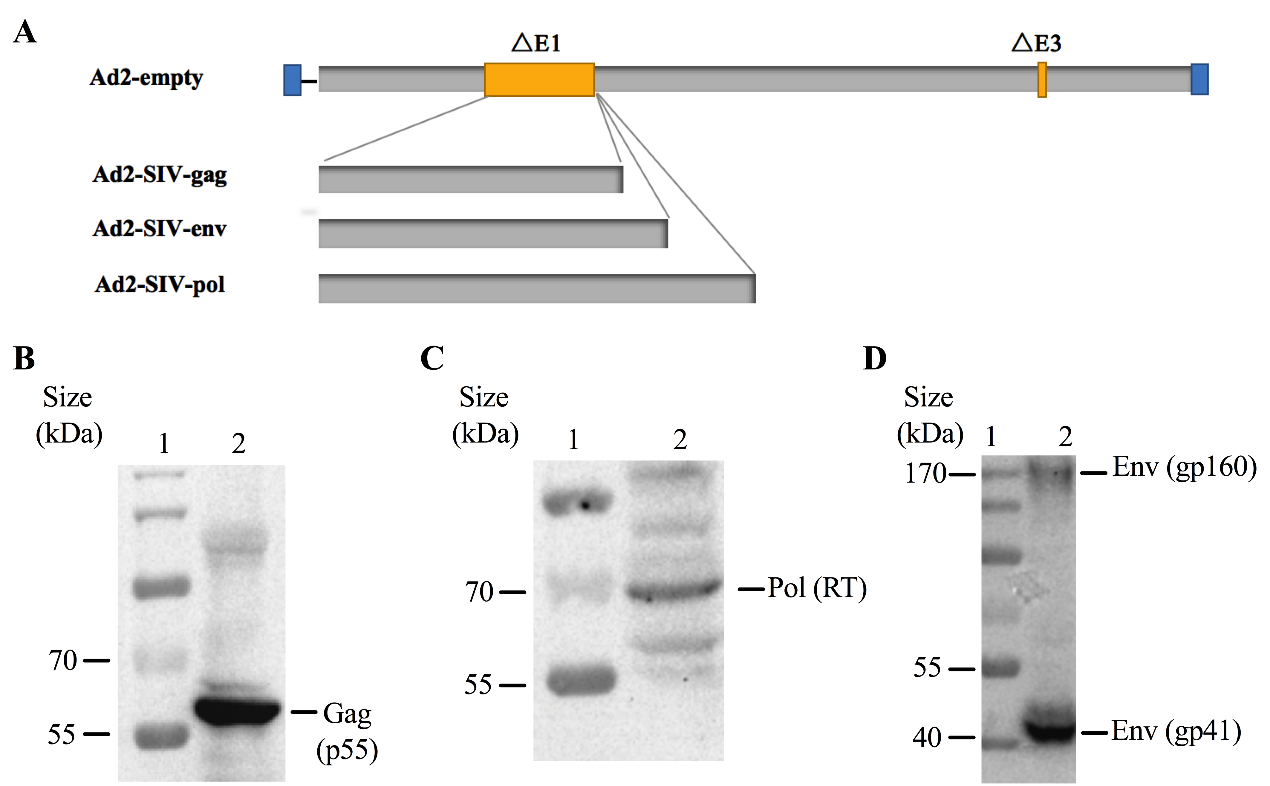
**

**Figure S2. Construction of Ad2-SIV-gag, Ad2-SIV-env, and Ad2-SIV-pol vaccines.**

1. Schematic representation of the construction of replication-incompetent recombinant adenovirus simian immunodeficiency virus (SIV) vaccines.

**(B-D)** Western blot analysis of the expression of Ad2-SIV-group-specific antigen (gag), Ad2-SIV-envelope (env), and Ad2-SIV-polymerase (pol). RT represents reverse transcriptase. Trex239 cells were infected with Ad2-SIV-gag, Ad2-SIV-env, and Ad2-SIV-pol, and verified by serum of an SIV-infected macaque.

**
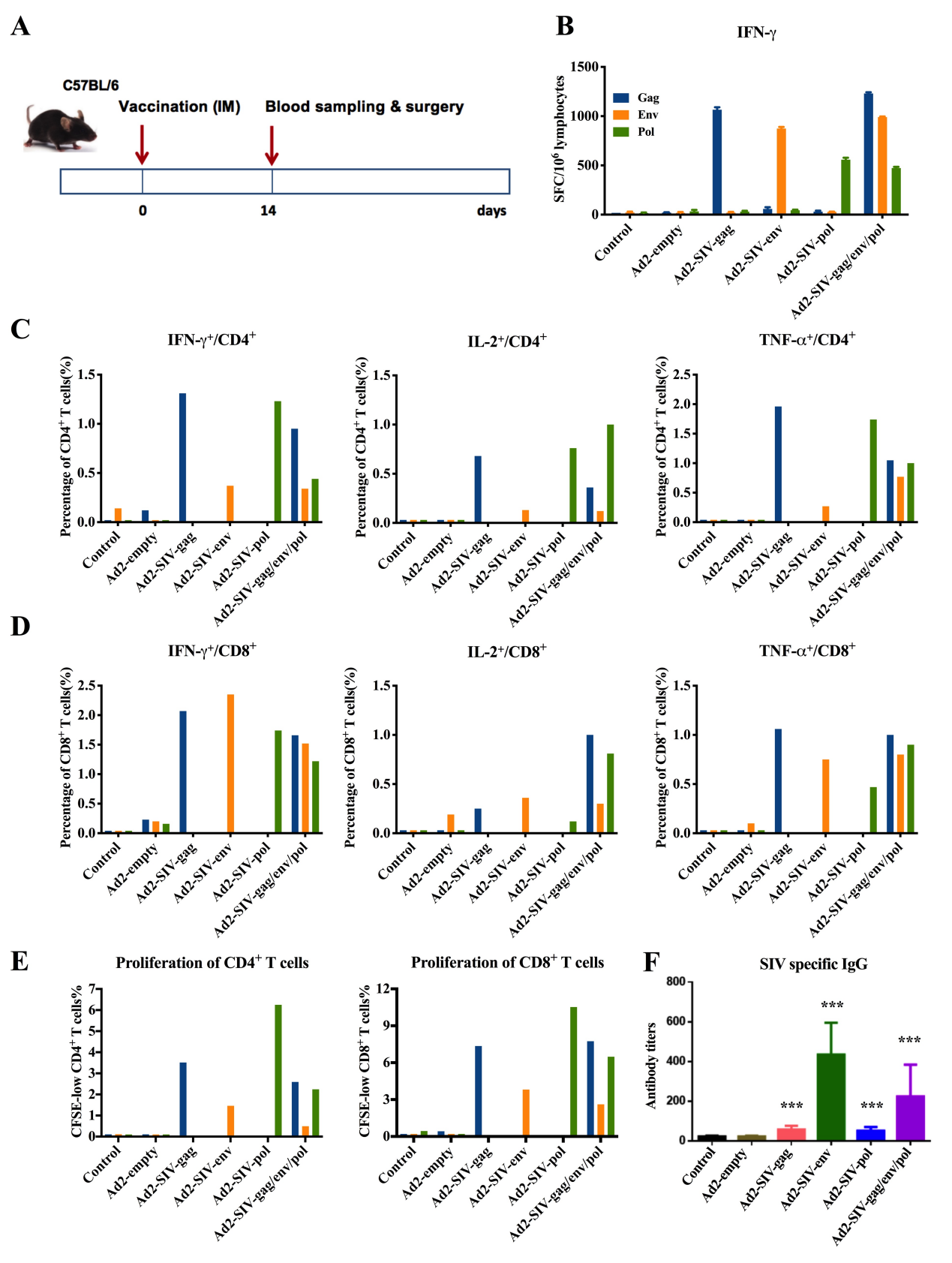
Figure S3. Evaluation of immunogenicity of adenovirus vectored SIV vaccines in mice.**

1. Schematic representation of the experimental design in mice.
2. Interferon (IFN)-γ enzyme-linked immunospot (ELISpot) assay for measuring antigen-specific T cell responses induced by SIV vaccines.
3. Intracellular cytokine staining (ICS) was used to measure the secretion of SIV-specific cytokines by CD4^+^ T cells.
4. ICS was used to measure the secretion of SIV-specific cytokines by CD8^+^ T cells.
5. SIV-specific proliferation of CD4^+^ T and CD8^+^ T cells was labeled with carboxyfluorescein diacetate succinimidyl ester (CFSE).
6. SIV-specific binding antibodies were measured by Enzyme-linked immunosorbent assay (ELISA). Data are represented as the mean ± SD. ***: p <0.001.

**
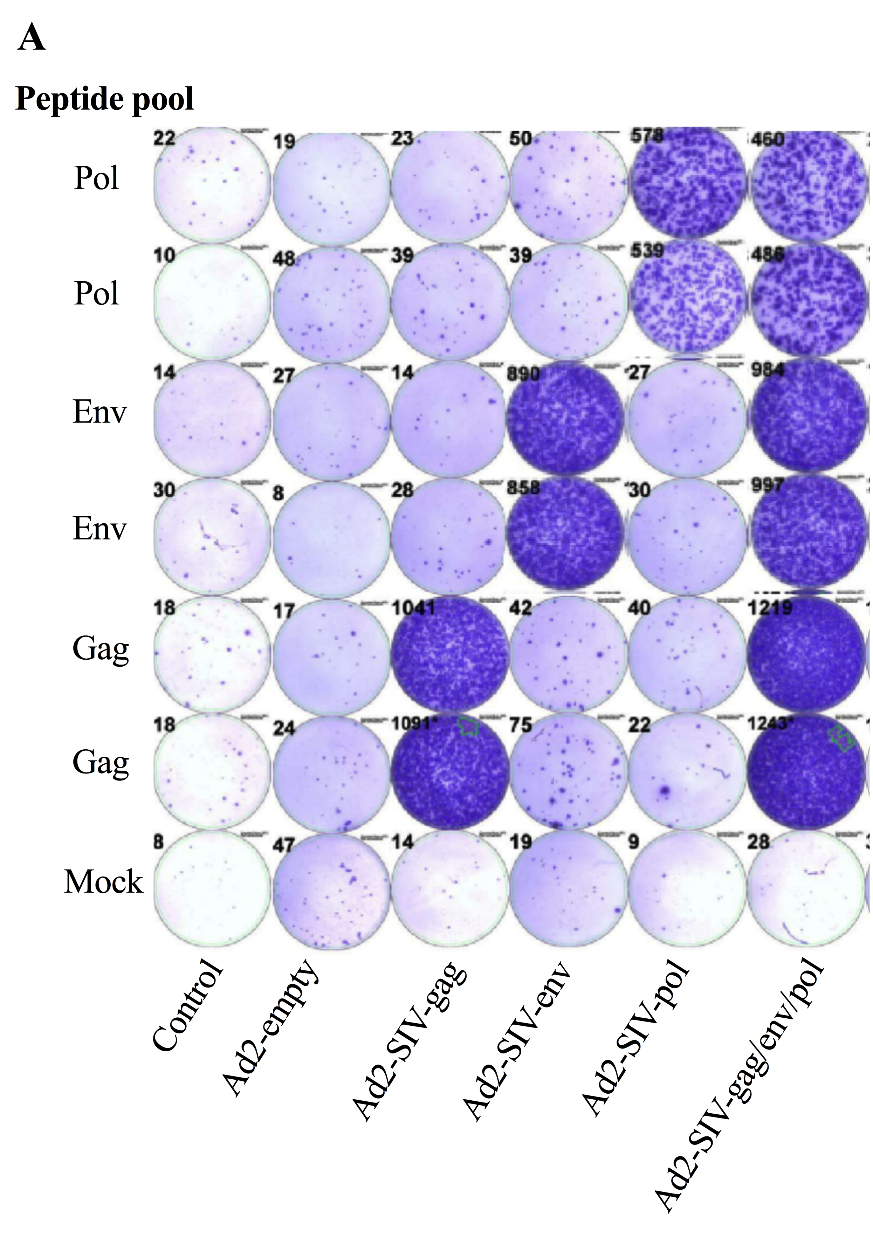
**

**Figure S4. Representative images of IFN-γ ELISpot**


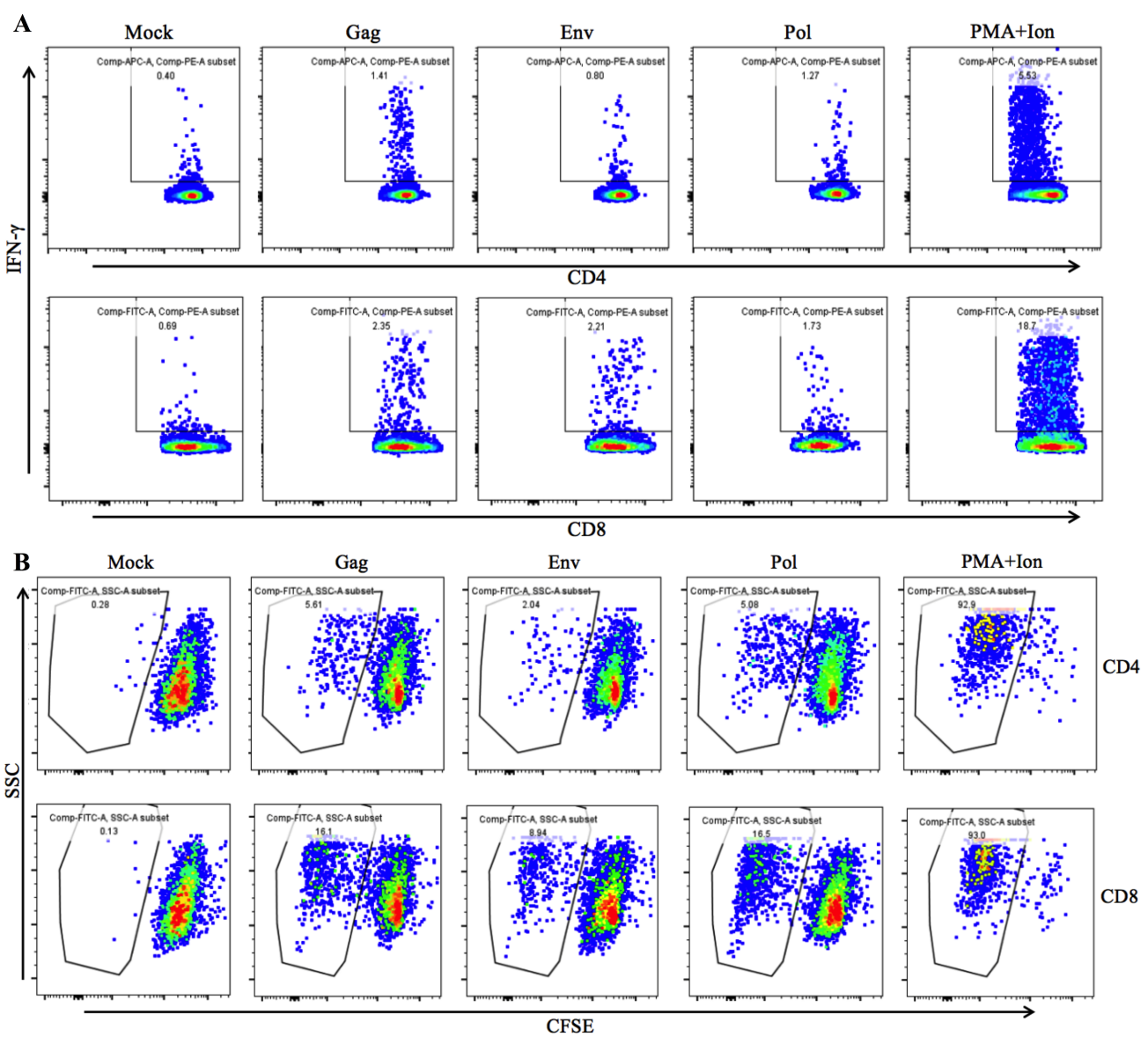


**Figure S5. Representative images of ICS and CFSE**

(A) The flow cytometry gating strategy for ICS.

(B) The flow cytometry gating strategy for CFSE.


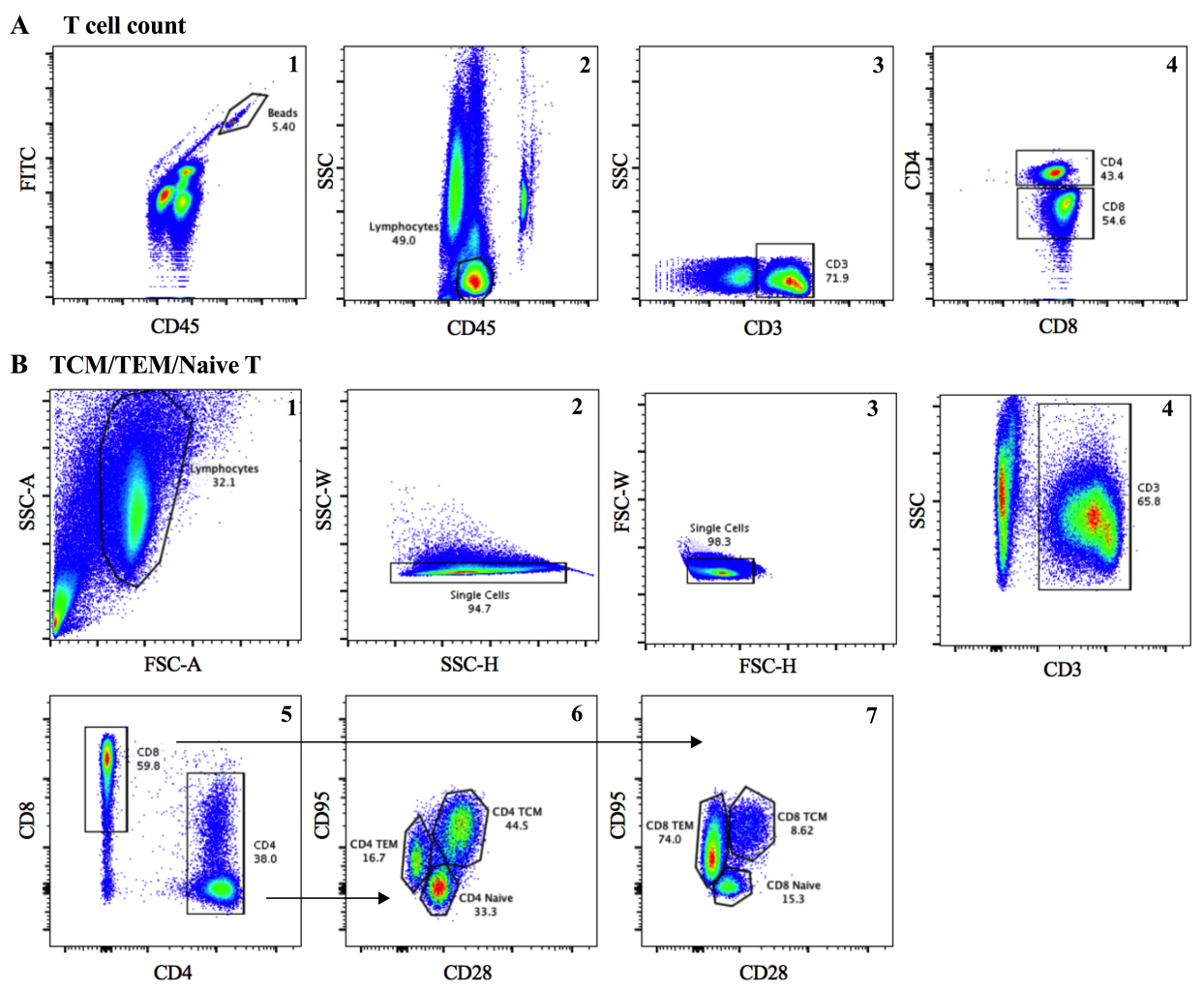


**Figure S6.** **The flow cytometry gating strategy.**

(A) The flow cytometry gating strategy for T cell counts.

(B) The flow cytometry gating strategy for effector memory T cells (TEM), central memory T cells (TCM), and naïve cells on CD4^+^ T cells and CD8^+^ T cells.


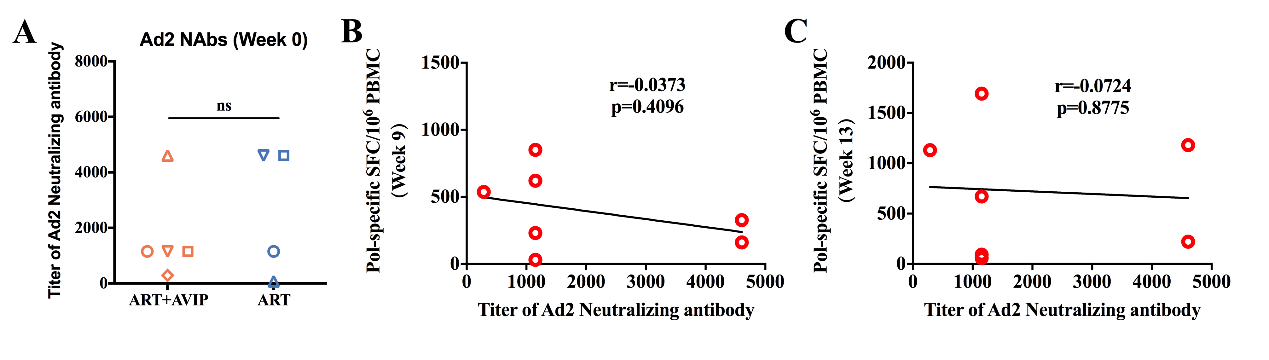


**Figure S7. Correlations between pre-existing anti-Ad2 NAbs versus the SIV-specific immune responses**

1. Pre-existing anti-Ad2 neutralizing antibodies (NAbs) at week 0.
2. Correlations between pre-existing anti-Ad2 NAbs versus the Pol-specific T cell response at week 9.
3. Correlations between pre-existing anti-Ad2 NAbs versus the Pol-specific T cell response at week 13.

**Figure S8. The expression of PD-1 on CD8^+^ T cells in SIV-infected macaques.**

1. The percentage of programmed cell death protein 1 (PD-1)^+^/CD8^+^ T cells was compared at different time points in AVIP+ART group and antiretroviral therapy (ART) group macaques.
2. Fold change of PD-1^+^/CD8^+^ T cell percentage at day 11 and 28 relative to day 0 in SIV-infected macaques.

**Table S1. Vaccination in mice**

| Group | Vaccine | Dose (vp/mouse) |
| --- | --- | --- |
| 1 | Control (PBS) | 100 μL |
| 2 | Ad2-empty | 1×10^10^ |
| 3 | Ad2-SIV-gag | 1×10^10^ |
| 4 | Ad2-SIV-env | 1×10^10^ |
| 5 | Ad2-SIV-pol | 1×10^10^ |
| 6 | Ad2-SIVgag/env/pol | 1×10^10^ + 1×10^10^ + 1×10^10^ |

Ad: adenovirus; SIV: simian immunodeficiency virus; gag: group-specific antigen; env: envelope; pol: polymerase; vp: viral particles.

**Table S2. Information of rhesus macaques**

| **groups** | **ID** | **Gender** | **Weight (kg)** | **Age (year)** | **Ad2 NAbs** | **SIV-specific IFN-γ ELISpot**  **SFCs** |
| --- | --- | --- | --- | --- | --- | --- |
| AVIP+ART | #1 | male | 9.58 | 14 | 1152 | 0 |
|  | #2 | male | 13.02 | 6 | 1152 | 0 |
|  | #3 | male | 9.2 | 6 | >4608 | 0 |
|  | #4 | female | 7.68 | 5 | 1152 | 0 |
|  | #5 | male | 9.96 | 6 | 288 | 0 |
| ART | #6 | male | 9.76 | 6 | 1152 | 0 |
|  | #7 | male | 10.86 | 6 | 4608 | 0 |
|  | #8 | female | 7.14 | 16 | 4608 | 0 |
|  | #9 | male | 16.98 | 6 | 72 | 0 |

AVIP: adenovirus vector-infected peripheral blood mononuclear cells; ART: antiretroviral therapy; NAbs: neutralizing antibodies; IFN-γ ELISpot: interferon (IFN)-γ enzyme-linked immunospot; SFCs: spot-forming cells.

**Table S3. Antibody information for flow cytometry analysis**

| **Name** | **Clone** | **Cat. No.** | **Brand** |
| --- | --- | --- | --- |
| FITC mouse Anti-Human CD4 | L200 | 550628 | BD Biosciences |
| PE-CF594 mouse Anti-Human CD4 | L200 | 562402 | BD Biosciences |
| BV605 Mouse Anti-Human CD4 | RPA-T4 | 562658 | BD Biosciences |
| APC mouse Anti-Human CD3 | SP34-2 | 557597 | BD Biosciences |
| Pacific Blue mouse Anti-Human CD3 | SP34-2 | 558124 | BD Biosciences |
| APC-cy7 mouse Anti-Human CD8 | RPA-T8 | 557760 | BD Biosciences |
| PercP mouse Anti-Human CD8 | SK1 | 347314 | BD Biosciences |
| PE mouse Anti-Human CD45 | HI30 | 560975 | BD Biosciences |
| FITC mouse Anti-Human CD28 | CD28.2 | 556621 | BD Biosciences |
| PE-cy5 mouse Anti-Human CD95 | 12X2 | 559773 | BD Biosciences |
| PE-eFluor 610 Anti-Human CD279 (PD1) | eBioJ105 | 61-2799-41 | eBioscience |
| FITC Anti-Human CD38 | AT-1 | 60131FI | Stem Cell |
| FITC Rat Anti-Mouse CD8α | 53-6.7 | 553030 | BD Biosciences |
| APC Rat Anti-Mouse CD4 | RM4-5 | 553051 | BD Biosciences |
| Percp-Cy5.5 Hamster Anti-Mouse CD3e | 145-2C11 | 551163 | BD Biosciences |
| PE Rat Anti-Mouse IFN-γ | XMG1.2 | 554412 | BD Biosciences |
| APC Rat Anti-Mouse IL-2 | JES6-5H4 | 554429 | BD Biosciences |
| PE-Cy7 Rat Anti-Mouse TNF | MP6-XT22 | 557644 | BD Biosciences |
| PE Rat Anti-Mouse CD8α | 53-6.7 | 553033 | BD Biosciences |
